# Effectiveness and Cost-Effectiveness of Internet-Based Cognitive Behavioral Therapy for Insomnia in Clinical Settings

**DOI:** 10.3389/fpsyt.2020.00838

**Published:** 2020-08-20

**Authors:** Polina Pchelina, Mikhail Poluektov, Thomas Berger, Tobias Krieger, Simone B. Duss, Claudio Bassetti

**Affiliations:** ^1^Institute of Psychology, University of Bern, Bern, Switzerland; ^2^Sleep Medicine Department, University Clinical Hospital No3, I.M. Sechenov First Moscow State Medical University, Moscow, Russia; ^3^Sleep-Wake-Epilepsy-Center, Department of Neurology, Inselspital, Bern University Hospital, University Bern, Bern, Switzerland

**Keywords:** sleep disorders, chronic insomnia, internet-based cognitive-behavioral therapy for insomnia, cost-effectiveness, outcome predictors

## Abstract

**Background:**

Internet-delivered cognitive-behavior treatment for insomnia (iCBT-I) has the potential to fill the gap created by the discrepancy between insomnia cases and number of trained professionals. Although the effectiveness of this method was proven in multiple studies conducted in research settings, its feasibility in routine care is still unclear. Predictors, mediators, and moderators of treatment effect remain uncertain since previous studies often give contradictory results. The present study aims to investigate clinical effectiveness and cost-effectiveness of an internet-based CBT-I program Sleepsy in comparison with care as usual (CAU) among patients with CI recruited from clinical settings. Baseline data will be further analyzed to find predictors of treatment outcome.

**Methods/Design:**

The proposed study is a parallel-group randomized controlled trial comparing CAU plus iCBT-I with CAU in a clinical setting. One hundred ten participants will be referred from the medical doctors in Moscow. Both groups will have access to CAU, which corresponds to the treatment prescribed by the referring doctor. Patients of the first group will additionally get access to the iCBT-I program with the opportunity to contact a specialist (guidance on request) in a secured environment. The primary outcome is insomnia severity change from pre- to post-treatment. Secondary outcomes include change of subjective sleep characteristics, life quality, fatigue, daytime sleepiness, comorbid affective disorders, dysfunctional beliefs about sleep, sleep hygiene, healthcare consumption, productivity losses, and longer term outcomes at 3 months follow-up. Predictor analysis will include baseline scores of the aforementioned outcomes along with treatment expectancies and personality traits.

**Discussion:**

The proposed study is one of the first studies evaluating whether iCBT-I also works in routine care. We expect that recruitment of the participants let us determine the target group more precisely and exclude health problems interfering with treatment. Using CAU as control condition may result in a loss of power to detect a meaningful difference. Nevertheless, this approach is reasonable since it reconstructs the clinical situation faced by practicing doctors.

## Introduction

Chronic insomnia (CI) is characterized by subjective perception of sleep complaints including difficulties initiating sleep, nighttime awakening, and disability to get back to sleep at night, non-restorative sleep, waking up too early in the morning, associated with any daytime repercussions, occurring 3 nights per week or more and persisting not less than 3 months (1). The burden of CI is high, with point prevalence ranging between 6% and 10% (2–4). Apart from deterioration in the quality of life of each patient, their families, and friends, CI imposes also a non-negligible social and economic burden. This burden includes resources used for treatment, as well as reduced or lost work productivity. CI impacts labor by means of time, efficiency, and results in productive work activities losses and may even lead to loss of employment. In 2011, estimated individual losses due to insomnia in the USA were 11.3 days of lost work performance per year, which equals $2,280 per year (5).

The prevailing “3P” model characterizes insomnia as the product of various factors that can be grouped as follows: predisposing, precipitating, and perpetuating (6). Insomnia more likely affects people with a certain vulnerability such as perfectionism, high concerns about sleep and health in general, external locus of control, and poor sleep habits. A stressful life event often causes an acute form of insomnia that mostly resolves without treatment. The chronic course of the disease is maintained by perpetuating factors, foremost of which are dysfunctional cognitions about the nature of sleep and causes of insomnia. These cognitions usually result in overestimation of sleep disturbances, channel attention on any evidence of their consequences, and entail maladaptive behavior, which is another important perpetuating factor. Since the etiology of insomnia is mostly multifactorial, the treatment of choice is also a multicomponent treatment, as is cognitive-behavioral therapy for insomnia (CBT-I) (7, 8). CBT-I consists of *sleep restriction*, *stimulus control*, various *relaxation techniques*, *sleep hygiene education*, and *cognitive restructuring* of maladaptive beliefs and concerns about sleep. Although particular treatment elements have shown its effectiveness, the multimodal approach has the highest level of evidence for the management of CI (9–11). The therapeutic effects of CBT-I are similar to or higher (especially at long-term) than those of hypnotic drugs (12). Nevertheless, time-consuming treatment courses and the lack of trained CBT-I clinicians cause difficulties in the implementation of these methods. Thus, developing innovative automatized and remote methods of performing CBT-I, such as internet-delivered CBT-I (iCBT-I), is of significant relevance.

In direct comparisons, individual face-to-face CBT-I showed better outcomes than internet-based CBT-I (iCBT-I) (13, 14). Comparison of iCBT-I with group CBT-I indicated that both treatment approaches are equally efficient (15). Furthermore, a meta-analysis reports a large within-group effect size for iCBT-I (Hedges’s *g* = 0.86) for self-reported insomnia severity (16). This effect size was not found to be significantly different then effect sizes in individual or group CBT-I (17, 18). A number of studies have shown the efficacy of iCBT-I in comparison with control groups receiving less active treatments (i.e., wait-list, sleep education, etc.) as summarized in several meta-analyses (19–21). The reported between group effect size for Insomnia Severity Index was large: Cohen’s d = **–**0.86, 95% CI –1.18, –0.53 (21). Since the overwhelming majority of iCBT-I randomized controlled trials (RCTs) were conducted in research settings with recruitment *via* internet and advertisement, it is unclear if the promising results from efficacy studies can be transferred to routine clinical practice. This aspect is essential because medical examination ensures proper diagnostics and patients’ safety. But practice shows that physicians usually lack time to provide regular CBT support in routine practice and tend to prescribe pharmacological treatment (22). The introduction of online nonpharmacological treatment approaches such as iCBT-I would fill the gap of feasible, effective and safe treatment methods.

The integration of a new method into clinical routine should consider clinical effectiveness, practicability, and cost-effectiveness. iCBT-I has shown cost-effectiveness in comparison with a wait-list for adults and adolescents (7, 23, 24). In this regard, more studies of iCBT-I cost-effectiveness, involving both the treatment and social costs, are needed to optimize patient management.

A reasonable way to improve the clinical effectiveness and cost-effectiveness is determining the target group of patients that benefits most of iCBT-I. As there is not much knowledge about predictors of iCBT-I treatment efficacy, in this trial, we will deliberately analyze predictors of CBT-I, which may also be relevant in iCBT-I. Although demographic variables (income, race, sex, age, and education) are analyzed in the majority of studies, there is no clear evidence that they predict treatment effect. There are studies showing better effects of iCBT-I and CBT-I in the elderly (25–27) and otherwise (28, 29). Furthermore, comorbid depression and anxiety are thought to complicate insomnia treatment and predict worse response to iCBT-I or CBT-I; however, existing data are discordant (25, 26, 30–32).

When analysis comes to insomnia as such, higher insomnia severity and lower quality of life are expected to increase patient’s motivation to adhere to treatment and be a predictor of treatment outcome as shown in studies on CBT-I (25, 32, 33). On the other hand, insomnia severity may mask comorbidities or atypical types of insomnia that complicate treatment (34). This factor is often related to objective and subjective sleep parameters. The idea that short sleep duration diminishes the effect of CBT-I was first raised in 2009 and was later confirmed in several studies where longer total sleep time predicted better response for CBT-I while shorter sleep time was associated with fewer improvements (32, 35, 36). It is further evident to assume that different sleep complaints patterns may be outcome predictors since they reflect various cognitive behavioral and biological factors. For example, our unpublished results showed that the absence of sleep onset problems, night awakenings, and more frequent early awakenings predicted lower insomnia severity after CBT-I (37). Generally speaking, the influence of age, comorbid psychological/psychiatric problems, and sleep characteristics on the effectiveness of CBT-I remains unclear and needs further investigation.

Unlike the aforementioned factors, there are no doubts that the efficacy of CBT-I is related to the severity of maladaptive cognitions and behavior. That was shown both for iCBT-I (38, 39) and CBT-I (25, 29, 30, 40). Coincidently alcohol, tobacco, and drug use negatively correlate with improvement after CBT-I (41).

Another critical factor is treatment adherence and should therefore be analyzed within research of treatment effectiveness (39). According to a meta-analysis, the adherence to iCBT-I, evaluated in two ways: self-reports and amount of participants who completed all sessions is around 50% (39). Even that low level is achieved under specific conditions, which are modular structure of the program, updating once a week, lasting for 10 weeks, including interaction with the system, a counselor, and peers (42). Studies of adherence and dropout from iCBT-I and CBT-I programs identify lower socioeconomic status, longer baseline total sleep time (TST), lower insomnia severity, and higher levels of depression and anxiety as predictors of dropout (43–45). These results may indicate that some participants who dropped out of insomnia treatment have lower demand for insomnia treatment and need more specific treatment of the comorbid disorder (depression and or anxiety).

The aim of the proposed RCT is to investigate clinical effectiveness and cost-effectiveness of the internet-based CBT-I program *Sleepsy* in comparison with care as usual (CAU) among patients with CI recruited from clinical settings. To our knowledge, this is one of the first studies considering iCBT-I in clinical settings. We hypothesize that the iCBT-I program would be superior to CAU with regard to effectiveness and cost-effectiveness. An equally important aim is to investigate predictors of treatment outcome by analysis of baseline characteristics.

Although Russia remains the largest country in the world by area with a population exceeding 144 million, the proposed study will be the first trial of online CBT-I, which makes it even more important. Due to unique aspects of culture, history, and different psychology traditions, CBT-I remains unknown and unavailable to the majority of Russian patients (46). Practice shows that general practitioners and medical doctors commonly lack time and knowledge about CBT-I. Furthermore, there is a shortage of skilled psychotherapists experienced in CBT-I and mental health practitioners (8.5 psychiatrists and 4.6 psychologists per 10,000 inhabitants in 2015) (47).

## Methods and Design

### Design

**Figure 1:**
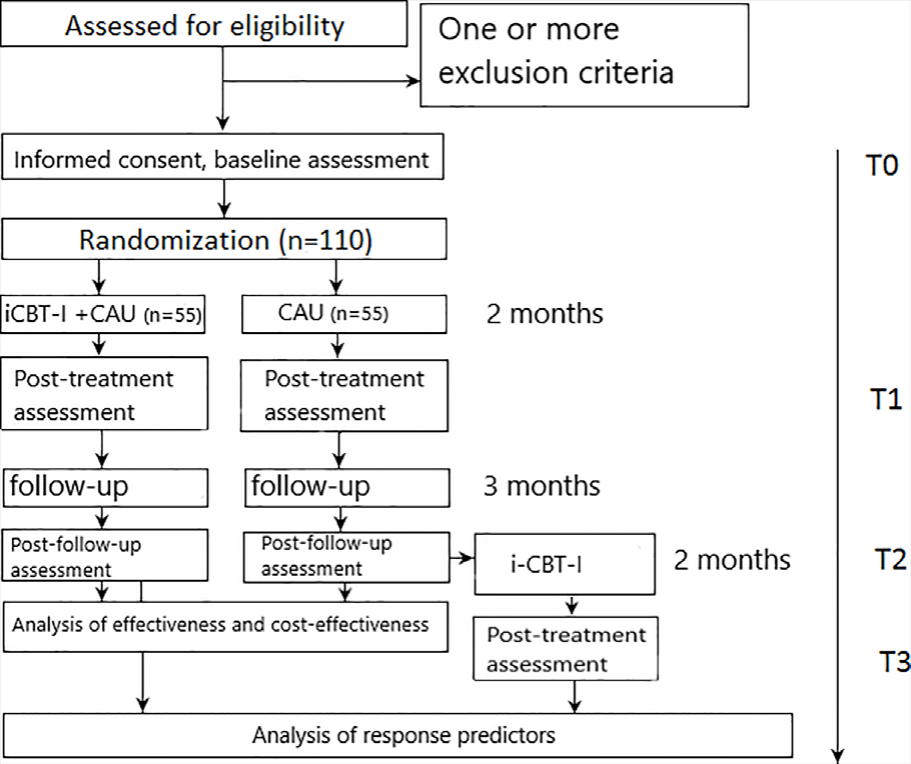
Participants flow diagram.

The study design is reported in line with the CONSORT 2010 statement (Consolidated Standards of Reporting Trials) (48) and the SPIRIT 2013 Statement (Standard Protocol Items: Recommendations for Interventional Trials) (49). The proposed study is a one-center parallel-group add-on superiority RCT comparing an active treatment condition (iCBT-I plus CAU) to CAU alone. Both groups will have access to CAU. Interventions that are part of CAU may be assigned within the first visit to a referring doctor or at any point of the study on a next doctor visit. All concurrently applied treatments will be assessed repeatedly by self-report. Assessments will be taken at 8-weeks (post-treatment assessment) and at the 5-month follow-up (5 months after randomization) (see **Figure 1**). Participants in the control group will get access to the iCBT-I program after the 5-month follow-up (3 months after post-assessment) provided they completed the follow-up assessment and still fulfill eligibility criteria (which will be assessed by a structured diagnostic interview conducted by phone by a medical doctor). In this group, an additional assessment will be conducted eight weeks after providing access to the CBT-I program to enlarge the sample for the analysis of outcome predictors. The study design is shown in **Figure 1**.

The study has been approved by the local ethics committee of the I.M. Sechenov Moscow Medical University (No. 03-20/19.02.2020) and has been prospectively registered in ClinicalTrials.gov Protocol Registration and Results System (Identifier: NCT04300218 21.04.2020).

### Participants

Patients with CI will be recruited through referrals from health care practitioners, i.e., neurologists specialized in somnology in Moscow (Sleep medicine department, University clinic 3, Sechenov First Moscow State Medical University, Moscow). Potential participants will be screened for inclusion and exclusion criteria during the primary or secondary visit at the University clinical department. In case of uncertainties, cases will be discussed with the principal investigator on site. Participation in the study requires health care practitioners to send a signed document stating that (1) they have seen/examined the patient, (2) they confirm CI diagnosis, and (3) that none of the exclusion criteria preventing participation in the study are present.

The referring specialists will explain to each participant the nature of the study, its purpose, the procedures involved, the expected duration, the potential risks and benefits and any discomfort it may entail, voluntary participation, withdrawal from the study at any time and that withdrawal of consent will not affect his or her subsequent medical assistance and treatment. After eligibility assessment, potential participants will receive a participant information sheet and a consent form. The informed consent (IC) is compiled in accordance with the National Standard of the Russian Federation for Clinical investigations and good clinical practice (GOST R ISO 14155-2014) for the creation of an IC for information transfer (50). Each participant will have seven days to decide whether to participate or not. After returning the signed IC, each participant will receive a copy of the IC signed by the authorized investigator.

#### Criteria for Inclusion

- Age between 18 and 80 years
- Sleep disorder matching CI criteria (International Classification of Sleep Disorders-3)—assessed by clinical judgement
- Ability to follow the procedures of the study, fluent Russian language, good access to internet—assessed by self-report

### Exclusion Criteria

- Presence of dementia (identified by history or a score < 25 on the Folstein Mini Mental Status Exam)—assessed by clinical judgement.
- Severe depression or severe anxiety as measured with the Beck Depression Inventory (BDI-II; score > 28) and the Beck Anxiety Inventory (BAI; score > 25)—assessed by the questionnaires.
- History of severe psychiatric comorbidities other than anxiety and depression (bipolar disorders, psychotic disorders) or substance use disorder—assessed by self-report and clinical history.
- Untreated severe obstructive sleep apnea syndrome (apnea-hypopnea index [AHI] > 15), restless legs syndrome (movement index with arousal > 15 per hour) or other sleep disorders affecting night sleep—assessed by clinical judgement and clinical history.
- Pregnancy, lactation—assessed by self-report.
- Having a serious somatic condition or brain disorders (stroke, Parkinson’s disease, etc.) preventing further participation—assessed by self-report.
- Having high suicidality risk—assessed by clinical judgement, high total BDI-II score (>28) or score >1 on a BDI-II of suicidality subscale.

### Randomization and Allocation Concealment

After signing the IC form, participants will receive an e-mail with the link to the Qualtrics Survey Software (Qualtrics, Provo, UT, USA), where they complete baseline assessment and receive the individual allocation code. On completion of baseline measures, subjects will be automatically assigned by the randomization function of Qualtrics Survey Software to either iCBT-I plus CAU or CAU group using single block randomization. Neither participants nor referring specialist nor the research team have foreknowledge and/or control over randomization and the allocation procedure. Following randomization, the participants will receive an automated email regarding their group allocation and a description of future steps. In case they were allocated to the treatment group, their individual code will provide an access to the iCBT-I program.

### Blinding

The research team, except the trial coordinator, will be blind to allocation. Coding allocation form, along with the participant’s real name and address noted in the IC form, will be stored in the password-protected computer of the trial coordinator. Participants will not be blind to treatment allocation and will receive information about their randomization outcome *via* email. Questionnaires and sleep diaries will be completed entirely online under the participant’s individual code.

### Assessment Points

Assessments will take place at weeks 0 (T0 - baseline), 8 (T1 - post-treatment), and 20 (T2 - follow-up) for both groups and additionally at week 28 (T3 - post-treatment) for the control group participants who will complete the follow-up assessment and subsequently offered the iCBT-I program.

### Intervention

Internet-based CBT-I (Sleepsy^©^) consists of interactive educational material divided into eight modules and explaining homeostatic and circadian mechanisms of human sleep regulation, the pathogenesis of CI, and its daytime consequences (**Table 1**). Modules provide the rationale for the main interventions of CBT-I: (1) sleep restriction with reducing time in bed to average self-reported sleep time over one week of sleep diary plus 30 min, with a minimum of 6 h and getting up at the same time every day, regardless of sleep duration; (2) stimulus control which implies to not go to bed unless sleepy and to not stay in bed unless asleep within 20 min; (3) napping is discouraged; (4) the cognitive techniques include dysfunctional belief restructuring (e.g., targeting unrealistic beliefs about sleep and the consequences of sleep loss). Information is presented in the form of short 10-min videos.

**Table 1:** Shows The Program Structure.

	iCBT-I Content
Module 1:	Introduction into the program structure and motivation for the program (statement that CBT-I is the treatment of choice for insomnia). Introduction of a sleep diary.
Module 2:	Psychoeducation: Information about sleep regulation and sleep hygiene.
Module 3:	Sleep Restriction: Stepwise reduction of time in bed (to min. 6 h) to increase sleep pressure based on the sleep diary that patients fill in since the beginning of module 1. If sleep efficiency is above 85%, participants can increase time in bed for 15 min.
Module 4:	Sleep hygiene and stimulus control: Information on sleep-inhibiting substances, sleep-wake-rhythm, evening rituals, physical exercise, etc., as well as information on the principle of stimulus control with the aim of re-associating the bed with sleep.
Module 5:	Progressive Muscle Relaxation (PMR) based on Jacobson with audio tape and written instruction to download.
Module 6:	Cognitive restructuring: Identifying, reexamining and changing dysfunctional thoughts about sleep and the consequences of insomnia.
Module 7:	Relapse prevention: what to do to prevent sleep disorders or when the sleep disturbances come back, when to seek medical help and what are the conditions of safe pharmacotherapy for insomnia.
Module 8:	Repetition and end.

The program includes a sleep diary in which participants insert data on their bedtime and waketime, sleep latency, total sleep time, and night awakenings. The data entered into the sleep diary are then presented in form of graphs. Furthermore, various exercises serve to put what is learnt into practice. Participants have time to repeat, deepen and apply modules, exercises and examples in their everyday life at their own pace, i.e., the participants are free to choose when and how often they process the modules and whether they process them in one piece or in several stages. Patients will get behavioral instructions based on their questionnaires, sleep diary, and answers in the interactive part of the program. A sleep window will be proposed depending on the preferred wake-up time and average total sleep time over the last 7 days. In order to avoid severe tiredness we have chosen more lenient minimal time in bed of 6 h. In the program, the caution to avoid severe sleep deprivation is made for elderly patients and for those who have duties in which drowsiness may be a danger to the patients themselves or others. These patients are recommended to flexibly extend sleep window. If the sleep diary indicates a sleep efficiency of 85% or higher for the previous week, the participant will be encouraged to add 15 min to the sleep window. If it does not reach the 85%, recommended sleep window will be decreased based on the preferred wake-up time and average total sleep time over the period of observation. If the sleep efficiency falls between 80% and 85% sleep window will remain stable otherwise (sleep efficiency > 85%) it can be extended by 15 min. Along with the general recommendations on sleep hygiene, participants will be encouraged to draw attention to the particular positions that gained highest score by Sleep hygiene index (i.e., reduce alcohol or coffee consumption close to bedtime if participant reports its frequent use) and by Dysfunctional Beliefs and Attitudes about Sleep Scale (DBAS; i.e., a participant who is highly concerned about consequences of insomnia indicated by a high score on the item “feel irritable, depressed, or anxious during the day, it is mostly because I did not sleep well the night before” will get recommendations to check if they have other reasons to be depressed and anxious regardless of insomnia).

All material will be delivered through the internet program and expected to be elaborated by the patients themselves but with the opportunity to contact a psychologist or somnologist within the program (guidance on request) in a secured environment. Furthermore, participants starting the program are encouraged to contact a specialist *via* feedback form if they face technical or methodological difficulties with the program have questions or suffer from negative effects of the intervention. The content of the program is based on an already established internet-based self-help program against insomnia that was already tested in a previous study (11).

### Outcome Measures

#### Primary Outcome Measures

The primary endpoint of the present study is the change of Insomnia Severity Index (ISI) from pre- to post-treatment and at follow-up. The ISI is a seven-item insomnia assessment tool, examining both nighttime and daytime aspects of insomnia disorder, and is sensitive to change CBT-I (51). The 5-point Likert scale is used to rate each item (e.g., 0 = no problem; 4 = very severe problem), yielding a total score ranging from 0 to 28. The total score is interpreted as follows: absence of insomnia (0–7); sub-threshold insomnia (8–14); moderate insomnia (15–21); and severe insomnia (22–28) (51). The response criterion is determined as decrease of the ISI score of more than 7 points compared to baseline (52). A Russian language adaptation of the test was validated and showed an internal consistency of 0.77 (53).

#### Secondary Outcome Measures

Secondary outcome measures include both disorder specific and transdiagnostic measures of sleep and quality of life, mood, behavior, cognitions, and personality traits. All the secondary endpoints, except Personality Inventory for DSM-5, will be evaluated as change from pre- to post-treatment and follow-up. Baseline scores will be included in analysis of predictors.

##### Subjective Sleep Characteristics

Subjective sense of sleep will find reflection in the sleep diary in such variables as sleep onset latency (SOL), total sleep time (TST), sleep efficiency (SE), calculated as TST/time in bed × 100, wake after sleep onset (WASO), and number of awakenings. SE may act as an additional response criteria since its increase of ≥10% was considered as such in previous works (54). In order to obtain these data we will ask control group participants to fill in online sleep diary in Qualtrics for one week starting at time-point T0 (after randomization), T1 and T2. iCBT-I + CAU group participants will fill it only at time-point T2 since online sleep diary is integrated into the iCBT-I program and we can use its data for the treatment and post-treatment period. We expect that data collected during the first week of intervention in iCBT-I + CAU group will not be compromised by treatment since educational material during the first week are introductive and motivational while active behavioral techniques start on the second week

##### Severity of Insomnia

Insomnia severity is important not only as primary measure of treatment effect but also as possible outcome predictor. To evaluate it, we will use baseline ISI score.

##### Daytime Impairment

Daytime symptoms of insomnia are often represented by sense of fatigue, sleepiness, and headaches. The fatigue severity will be evaluated by the Fatigue Severity Scale (FSS), a disorder non-specific 7-item scale which measures the severity of fatigue and its effect on a person’s activities and lifestyle. This scale demonstrates high internal consistency in patients with mental health problems with alpha = 0.93 (55).

##### Quality of Life

Short-form survey (SF-12) version 1.0 is a non-specific scale assessing quality of life by means of eight subscales (vitality, physical functioning, bodily pain, general health perceptions, physical role functioning, emotional role functioning, social role functioning, mental health) (56, 57). The score is a combination of a mental component score (MCS-12) and a physical component score (PCS-12). Result can be presented as MCS-12 and PCS-12 differences compared to the population average, measured in standard deviations. The referent PCS-12 and MCS-12 are both 50 points. It is commonly used to calculate quality-adjusted life years (QALYs), which is a measure of disease burden. It has internal consistency 0.69–0.70 as measured for adults with mental health conditions.

##### Sleepiness

Sleepiness as one of the most often reported symptoms of CI will be evaluated by the Epworth Sleepiness Scale (ESS) (58), a measure asking propensity for “dosing” in eight daytime situations from 0 = never to 3= very high propensity, yielding a total score ranging from 0 to 24 with normal score < 9.

##### Anxiety and Depressive Symptoms

Anxiety and depressive symptoms will be assessed with the Beck Anxiety Inventory (BAI) and the Beck Depression Inventory (BDI-II) (59, 60). Both of them comprise 21-question with a 4-point Likert scale and ranging answers from 0 to 3, yielding a total score ranging from 0 to 63. A BAI total score higher than 25 corresponds to severe anxiety, and a BDI cutoff higher than 28 indicates severe depression. The Russian validated version of the BDI-II has an internal consistency of.72 (61). English version of BAI has high internal consistency (Cronbach’s a = 0.92). Its Russian language version demonstrated high level of construct validity in comparison with other validated Russian language anxiety measures (62).

##### Behavioral Perpetuating Factors

One of the leading perpetuating factors of insomnia is poor sleep habits that can be assessed by the Sleep Hygiene Index, a 13-question questionnaire evaluating each item on a 5-point Likert scale, yielding a total score ranging from 13 to 65. Questionnaire internal consistency comprises 0.66. (63). We have translated it into Russian according to a forward and backward translation procedure.

##### Cognitive Perpetuating Factors

Equally important are cognitive perpetuating factors such as thoughts, feelings, and beliefs that may lead to maladaptive behavior affecting sleep. These factors will be assessed by the Sleep Locus of Control Questionnaire (SLC) (64) and Dysfunctional Beliefs and Attitudes About Sleep Scale (DBAS) (65). The SLC variant validated in Russia comprises 8 questions scored using 6-point Likert scale ranging each answer from 1 = strongly disagree; 6 = strongly agree, yielding a total score ranging from 8 to 64. The Russian adaptation of the scale has shown internal reliability of 0.41 (53). The DBAS was designed to identify and assess severity of various sleep and insomnia-related cognitions. It consists of 16 questions with a Likert scale ranging answers from 0 = strongly disagree to 10 = strongly agree with a total score ranging from 0 to 160. The Russian adaptation of the scale has high internal reliability of .86 (53).

##### Personal Traits of Participants

Description of personal characteristics may play an important role in predicting treatment outcome. For this purpose, we intend to use the Personality Inventory for DSM-5 faceted brief form (PID-5-FBF) 100-item self-report inventory designed to assess the 25 pathological personality trait facets and the five domains based on the dimensional trait model (DSM-5 Section III) (66). This psychodiagnostic instrument was created to address the criticisms of DSM-IV-TR categorical approach to personality disorders and later demonstrated high construct validity with other personality models. Items of the test are rated on a 4-point Likert scale from 0 (very false or often false) to 3 (very true or often true). English version has internal consistency from 0.72 to 0.96 (median = 0.86) for different facets. For translation the questionnaire we used forward and backward translation procedure.

##### Socioeconomic Factors

In order to evaluate the cost-effectiveness of iCBT-I, we will collect information concerning healthcare consumption and productivity losses for the study period using the Trimbos Questionnaire for Costs associated with Psychiatric illness (TiC-P) consisting of two parts. The first part consists of 14 questions on the volume of health care uptake including names, dosage, and frequency of use of medications for insomnia treatment; the second part is represented by the Short Form- Health and Labor Questionnaire (SF-HLQ), an instrument to collect data on productivity losses (presenteeism and absenteeism) due to health problems (67). Reliability of TiC-P is characterized with intra class correlation coefficient for test-retest measurements equal 0.83. Another reliability criterion of TiC-P, absolute agreement, reflecting consistency between patient-reported data and data from medical records ranges from 0.82 to 0.99 (67). For the proposed study questionnaire was translated into Russian.

#### Additional Measures

##### Adherence

During the intervention, we will assess adherence by recording of the number of completed modules, total time spent on the iCBT-I website, time spent on each module, number of completed sleep diaries and usage of the support. Time spent on the website will be measured by means login/logout data and by keeping an activity journal.

##### Success Expectancy

In order to analyze such important outcome predictor as patients beliefs about the expected treatment success and to evaluate between-group difference with regard to this factor, we include in baseline assessment one adapted question of Credibility/Expectancy Questionnaire: “At this point, how successfully do you think this treatment will be in reducing your insomnia symptoms?” at scale from 1 to 9. This question was chosen as well representing high correlation for both factors: credibility and expectancy, and most logically formulated for the intended purpose (68).

##### User Satisfaction

After completion of the iCBT-I course participants will be requested to fill in feedback questionnaire developed for this study. It includes 5-point Likert scale, from 1 (very poor/not at all useful) to 5 (very, good/very useful) on questions about satisfaction or dissatisfaction with the treatment, and open questions about possible negative effects of the intervention if any: deterioration of insomnia symptoms, adverse effects, novel symptoms; and about improvement suggestions, what participants like most, if they would recommend it to a friend with insomnia.

##### System Usability

The System Usability Scale (SUS) is the 10-item non-specific questionnaire used to collect a user’s subjective rating of a product’s (products, websites, applications, hardware, or software) usability and learnability (69, 70). Each item is scored on a scale of 0 (“strongly Disagree”) to 5 (“strongly Agree”).

### Assessment of Safety

We will assess depressive and anxiety symptoms with the BDI-II and BAI as a safety outcome at all times of data collection, i.e., as part of the initial screening, during and after the intervention, as well as at the follow-up measures. Severe depression and anxiety are defined as BDI-II scores > 28 and BAI scores > 25, accordingly. If scores are higher than these cutoffs at the time of the screening, the person concerned will not be admitted to participate in the study and will be referred to a suitable authority such as a psychotherapist, a psychiatric clinic, or a hospital. Should this occur during another time throughout the study, participants will also be given information on suitable institutions to turn to and asked to inform the contact person for emergencies named by them at the beginning of the study.

Furthermore, participants receive a weekly individualized automated e-mail during the intervention, acknowledging their progress and offering help if they encountered difficulties with the program. Persons who had to be directed to psychiatric/psychotherapeutic services will be contacted by the study team as soon as possible to assure that they are in professional care.

### Sample Size Calculation

We aim to detect a small to medium between-group effect size (Cohen’s d) of 0.35 at post-treatment. The chosen effect size is reasonable since CAU being an active alternative treatment may decrease between-group outcome differences and a smaller effect size was considered to be irrelevant from a clinical point of view (11). Based on α-level of.05 and a power of.95 using a single-level repeated measures ANOVA power analysis with G*Power (71), the sample size needed is approximately 55 participants per group and 110 for the total sample. Our secondary aim is to find outcome predictors within participants who complete iCBT-I. In order to enlarge this sample, participants from the CAU group will get access to the iCBT-I program after the follow-up period.

### Statistical Analysis

The main analyses will be conducted on the intention-to-treat sample. Clinical effects of treatment will be evaluated using mixed-model repeated-measures analyses where the pre-post comparisons of all outcome measures will include time as a within-group variable and the condition as a between-group variable. An incremental cost-effectiveness ratio (ICER) will be calculated to obtain the additional costs of iCBT-I in comparison with control condition. Cohen’s *d* will be reported for all within-between comparisons based on estimated means and the pooled standard deviation from the observed means at pre- and post-treatment. To test significance of the difference between the number of responders in each treatment condition, we will use χ^2^-test. Regression analyses will be used to identify predictors of treatment outcome. We will test models using Akaike/Bayesian Information Criteria to select an optimal model with predictors of treatment outcome.

## Discussion

The introduction of an internet-delivered approach into clinical practice of CI treatment is promising since it gives patients a choice of place and time for treatment and provides easier access to professional service for patients especially in view of huge demand for psychotherapists experienced in CBT-I in Russia. It also matches a stepped-care model of insomnia treatment, which imply that patients with uncomplicated CI may start treatment with more accessible and low-cost variants of CBT-I and “shift” to more complex ones with higher degree of involvement of specialists if needed (72). For instance, it has been shown that patients may benefit from bibliotherapy or brief behavioral therapy—methods which do not necessarily require trained psychotherapist (72). We assume that internet-delivered CBT-I is applicable for a subgroup of patients with uncomplicated insomnia, who are motivated for nonpharmacological treatment or remain symptomatic despite pharmacotherapy (73, 74). It is, however, noteworthy that online programs have to be safe and effective both from a clinical and an economic point of view. The present study aims to evaluate the clinical and the cost-effectiveness of the proposed program. As a secondary objective, we aim to obtain a portrait of patients who benefit from iCBT-I. This information will eventually give us cues to understanding insomnia in general and its subtypes in particular. Moreover, this information will give the opportunity to develop individualized therapeutic decision-making procedure to simplify insomnia treatment, improve patient adherence, and decrease of treatment costs.

To our knowledge, there is only one study of clinical effectiveness of iCBT for insomnia, in primary care, where patients were invited to participate in the study in two ways: during the general practitioner consultation or by means of the invitation letters referred to those patients who consulted for insomnia in the past year (75). Authors of this study report significant decrease of insomnia severity and improvement of sleep diary characteristics. However, this study did not take into account cost-effectiveness. The other study conducted in clinical settings used ISI and QALY as outcomes and reported significantly higher cost-effectiveness of iCBT-I compared with CAU (20).

Recruiting from primary care has at least two benefits. First, it allows us to determine the target group more precisely and exclude health problems that may interfere with treatment. Because we do not plan to exclude participants with stable or treated co-occurring medical or psychiatric conditions as well as participants with mild to moderate obstructive sleep apnea syndrome or periodic limbs movement syndrome, generalizability of the expected findings will be high. Second, it may be that patients, referred to iCBT-I directly from doctor’s office, have active and more severe sleep complaints, and therefore are more motivated to benefit from the treatment (32, 33, 76, 77). A lower dropout rate has previously been reported for an iCBT-I study conducted in primary care settings (75). These arguments let us rate recruitment in clinical settings as the key strength of the proposed study.

Several limitations of the study should be considered. CAU, being the active control condition, has disadvantages since it may compete with the experimental treatment narrowing differences between groups, and resulting in loss of power to detect a meaningful difference (78, 79). Moreover, CAU may interfere with iCBT-I in the experimental group, i.e., in participants who are prescribed benzodiazepines (prolonging sleep and enhancing daytime sleepiness) and trying to shorten their sleep window or avoid daytime naps at the same moment. Nevertheless, this choice of control condition is necessary to evaluate iCBT-I implementation in real clinical practice. An analysis of pharmaceutical agents used within routine care is necessary for evaluation of iCBT-I cost-effectiveness.

A further limitation of our study is that the follow-up period is rather short, given that the persistence of iCBT-I treatment effect remains questionable. A study of Sleep Healthy Using the Internet (SHUTi) CBT-I program proved maintenance of the treatment effect after an 18-month follow-up period (80, 81). Another study reports that the effects of iCBT-I were maintained for 36 months; however, insomnia severity in iCBT-I did not differ statistically from the control group at the end of the follow-up (82). One more limitation is a small sample size limiting power of predictors’ analysis. Given that this analysis is not the primary objective of the study, it will be exploratory in nature and aims at identifying possible predictors that then can be the focus in future research. Despite the aforementioned limitations, the proposed study is timely and pertinent, because the full and comprehensive evaluation of iCBT-I will let us introduce this approach into clinical practice, and facilitate insomnia treatment both at an individual and federal level.

## Dissemination

Results of this trial will be disseminated *via* peer-reviewed journal publications. Primary and secondary aims will be reported in a single publication. Other findings will be published separately. The results of this study will also be available on the ClinicalTrials.gov website when they become available.

## Ethics Statement

The studies involving human participants were reviewed and approved by Local Ethics Committee of the I.M. Sechenov Moscow Medical University. The patients/participants provided their written informed consent to participate in this study.

## Author Contributions

All authors contributed to the conception and design of the study: PP, MP, SD, and TK developed the intervention program and contributed to its description. MP and PP managed the participants’ recruitment. PP managed the flow of participants and the assessment process. MP, PP, and TB contributed to the sample size calculation. CB contributed to the eligibility criteria of the study. PP wrote the draft of the manuscript. All authors contributed to the article and approved the submitted version.

## Conflict of Interest

The ICBT program for insomnia used in this study was developed by the authors for research purposes but is delivered, from a technological standpoint, *via* a web-based software developed by ООО Техномаркт Ltd, a Russian private company. PP is working as an external consultant for the project of this company. The project targets counseling shift workers and has no connection to this manuscript.

The remaining authors declare that the research was conducted in the absence of any commercial or financial relationships that could be construed as a potential conflict of interest.
